# Hand Surgery Anaesthesia Innovations: Balancing Efficiency, Cost, and Comfort with WALANT, Ultrasound, and Emerging Adjuncts—A Narrative Review

**DOI:** 10.3390/jcm14176146

**Published:** 2025-08-30

**Authors:** Omar Shadid, Jennifer Novo, Raj Saini, Gianluca Marcaccini, Brett K. Sacks, Warren M. Rozen, Ishith Seth, Roberto Cuomo

**Affiliations:** 1 Faculty of Medicine and Surgery, The University of Melbourne, Melbourne 3051, Australia; 2 Faculty of Medicine and Surgery, University of Notre Dame, Sydney 2010, Australia; 3 Faculty of Health Sciences, Simon Fraser University, Burnaby, BC V5A1S6, Canada; 4 Department of Plastic and Reconstructive Surgery, University of Siena, 53100 Siena, Italy; 5 Department of Plastic and Reconstructive Surgery, Peninsula Health, Melbourne 3199, Australia; 6 Faculty of Medicine and Surgery, Peninsula Clinical School, Monash University, Melbourne 3199, Australia

**Keywords:** WALANT, ultrasound-guided nerve block, vapocoolant spray, hand surgery, ambulatory anaesthesia

## Abstract

**Background:** Hand surgery is increasingly transitioning from hospital operating theatres to outpatient settings, requiring anaesthetic methods that are efficient, cost-effective, and patient-centred. Traditional anaesthesia, such as general anaesthesia, poses challenges including prolonged recovery and physiological stress. Novel strategies, such as Wide-Awake Local Anaesthesia No Tourniquet (WALANT), ultrasound-guided distal nerve blocks, and adjunctive approaches (vapocoolant spray, patient-controlled regional analgesia, cryoanalgesia, jet injectors), have emerged to address these limitations. This narrative review consolidates current evidence regarding the efficacy, applicability, and economic implications of these evolving anaesthesia techniques. **Methods:** A literature search was conducted across MEDLINE, Embase, CENTRAL, and Scopus databases up to 1 June 2025. Inclusion criteria were English-language original studies on WALANT, vapocoolant sprays, ultrasound-guided distal nerve blocks, or emerging adjunctive anaesthesia methods applicable to hand and upper limb surgery. Exclusion criteria included non-English publications and those without original clinical data. Two independent reviewers screened and selected studies, ensuring relevance and methodological quality. **Results:** WALANT can provide high patient satisfaction, cost savings of 70–85%, and allow for real-time functional testing during surgery. Ultrasound-guided nerve blocks provided targeted analgesia, preserved elbow function, reduced the need for sedation, and improved perioperative efficiency. Adjuncts such as vapocoolant sprays significantly decreased needle-injection discomfort, offering quick and economical analgesia for superficial procedures. Other emerging adjuncts, including patient-controlled regional anaesthesia (PCRA), cryoanalgesia, and jet injectors, offered additional patient-tailored pain management options, although with higher resource demands. **Conclusions:** The review highlights the transformative potential of WALANT and adjunctive techniques to enhance efficiency, patient experience, and cost-effectiveness in hand surgery. Despite clear benefits, optimal application requires tailored patient selection, clinician familiarity, and consideration of procedure-specific demands.

## 1. Introduction

In North America, over half of the elective hand and wrist procedures are performed as day case interventions in ambulatory surgery centres, office-based suites and dedicated procedure rooms, with European centres rapidly following suit to meet growing demand and contain costs [1]. This shift from traditional operating theatres underscores an industry-wide move towards more efficient, patient-centred care in hand surgery. Large datasets demonstrate that low-risk procedures, such as carpal tunnel or trigger finger release, can be performed safely outside the main operating room, with complication rates comparable to or lower than those reported for hospital-based surgery, and with significantly lower payer expenditure [2]. This outpatient shift demands anaesthetic strategies that are efficient, economical and patient-centred.

Conventional anaesthetic approaches in hand surgery present several limitations. General anaesthesia ensures immobility but necessitates preoperative fasting, airway instrumentation, and extended postoperative recovery; factors that can result in physiological stress and increase utilisation of limited operating theatre resources. Proximal regional techniques, such as brachial plexus blocks, achieve complete limb anaesthesia; however, this comes at the expense of real-time functional testing and can often require an upper-arm tourniquet to maintain a bloodless field [3]. While effective, prolonged tourniquet inflation without sedation can lead to patient discomfort and restrict the feasibility of these blocks in ambulatory settings. Collectively, these challenges contribute to slower patient throughput and reduced satisfaction. In response, novel strategies have emerged.

Among the most prominent are WALANT and ultrasound-guided regional blocks. Each offers specific benefits across diverse clinical contexts and reflects broader goals to improve safety, reduce resource demands, and personalise care. WALANT enables surgery without sedation or tourniquet by infiltrating diluted local anaesthetic with epinephrine directly into the operative field [4,5]. This approach provides analgesia and haemostasis while allowing the patient to remain awake and actively participate during procedures such as tendon repair [5,6]. WALANT has gained popularity over the past twenty years as a safe and effective alternative to traditional anaesthesia methods, whilst also offering cost savings of 70–85% and high patient satisfaction [4].

Ultrasound-guided distal nerve blocks are increasingly used for hand and wrist surgery [7,8]. Historically, anaesthesia of the upper limb relied on proximal brachial plexus blocks (e.g., axillary or supraclavicular) or intravenous regional anaesthesia (IVRA), while local infiltration was limited to minor cases [8,9]. High-resolution ultrasound now enables the precise identification and blockade of the median, ulnar, and radial nerves at or below the elbow, thereby renewing interest in distal blocks for surgical and postoperative use [8,9,10]. These techniques reduce reliance on general anaesthesia and systemic medication.

As these techniques become more integrated, new adjuncts are also being explored to enhance flexibility and patient autonomy. Key innovations include vapocoolant spray, patient-controlled regional anaesthesia (PCRA), cryoanalgesia, and jet injection [11,12,13]. Vapocoolant anaesthesia, also known as “cold spray,” provides rapid, superficial analgesia through evaporative skin cooling [14,15]. Volatile agents, such as ethyl chloride, slow nerve conduction, producing near-instantaneous numbness of the skin [16,17]. Long used in minor procedures, they are increasingly applied in hand surgery to reduce discomfort during injections or superficial interventions. PCRA, on the other hand, facilitates patient-activated bolus dosing via perineural catheters [18]. Cryoanalgesia entails the application of targeted cold to disrupt nerve transmission over prolonged durations [19]. Jet injectors administer local anaesthetic transdermally, providing a needle-free alternative for superficial procedures [13].

Collectively, these strategies reduce reliance on systemic opioids and sedatives, particularly in minor procedures and ambulatory settings, contributing to shorter recovery times and a reduced incidence of opioid-related adverse effects. While individual techniques have shown benefits in specific contexts, clinicians lack an integrated framework for selecting among them. This narrative review consolidates current evidence on evolving anaesthetic strategies in hand surgery and aims to clarify their comparative indications, benefits, and limitations to support evidence-informed decision-making by surgeons, anaesthetists, and perioperative teams.

## 2. Methods

We conducted a narrative review informed by a structured literature search to examine the effectiveness, applicability, and economic implications of emerging anaesthesia techniques in hand surgery. The search strategy was designed to be systematic in approach, but the review itself was narrative in scope, aiming to provide a comprehensive synthesis rather than an exhaustive systematic analysis.

The literature search was carried out across four electronic databases: MEDLINE (via OVID), Embase (via OVID), the Cochrane Register of Trials (CENTRAL), and Scopus. Searches covered the entire available date range of each database up to 1 June 2025. Boolean operators “AND”/”OR” were combined to maximise sensitivity and specificity. Key search terms included: “WALANT” (Wide Awake Local Anaesthesia No Tourniquet) and its expanded form; “cold spray” (or “vapocoolant anaesthesia”); “ultrasound-guided anaesthesia”; “hand surgery” combined with “anaesthesia”; and “local anaesthesia techniques” combined with “upper limb surgery.” To broaden the search, we also included terms for related adjuncts such as “brachial plexus block,” “patient-controlled anaesthesia,” “cryoanalgesia,” and “jet injection.”

The search was restricted to studies involving human participants and published in the English language. Two authors (OS and RS) independently cross-referenced the abstracts. The final list of studies was jointly reviewed for relevance to the proposed topic. Eligible papers met the following criteria: (1) investigated anaesthesia techniques in hand or upper limb surgery; (2) examined WALANT, cold spray/vapocoolant anaesthesia, ultrasound-guided regional blocks, or other novel anaesthesia techniques relevant to hand surgery; (3) constituted original research, including randomised controlled trials, cohort studies, case–control studies, or case series; and/or (4) included landmark trials, large case series, or studies providing robust economic data.

Studies were excluded if they: (1) were not in English or lacked a high-quality adjunct translation; (2) did not focus on anaesthetic techniques for hand or upper limb surgical procedures; (3) lacked original clinical data (e.g., reviews, expert opinions, editorials, commentaries, or conference abstracts without accessible data); or (4) did not meet the thresholds for landmark trials, large case series, or relevant economic analyses where applicable.

## 3. Discussion

### 3.1. Current Clinical Applications

#### 3.1.1. WALANT

WALANT has become a versatile and widely adopted technique in hand surgery. Initially applied to minor soft tissue procedures, it is now used in more complex surgeries involving tendons, nerves, bones, and reconstruction due to its ability to provide haemostasis and enable intraoperative patient participation [20].

Its utility is especially evident in flexor and extensor tendon repairs (zones II and III), where active patient movement allows real-time testing of glide, suture security, and pulley function [21,22]. This intraoperative feedback reduces the risk of rupture and enables immediate adjustments. Surgeons can also assess bowstringing and perform more aggressive pulley venting safely [23]. Observing finger movement has been linked to improved adherence to therapy [23,24].

WALANT is also commonly used for carpal tunnel release (CTR), trigger finger release, Dupuytren’s fasciectomy, and ganglion excision. In some Canadian centres, over 95% of CTRs now occur outside traditional theatres [25]. Surgeons can confirm decompression visually, improving outcomes [26,27]. Dupuytren’s surgery shows similar benefits with high satisfaction and low complication rates [28,29,30]. Its role has expanded to include fracture fixation. Phalangeal and metacarpal fractures are often repaired using K-wires or plates under wide-awake conditions. For distal radius fractures, WALANT-based ORIF yields similar outcomes to general anaesthesia, with lower cost and no admission [10,31,32]. Even advanced procedures, such as small joint arthroplasty, nerve repair, tendon transfers, and flap reconstructions, are increasingly performed under WALANT [33,34,35,36]. Surgeons can dynamically assess functional outcomes intraoperatively and optimise tensioning [33,36]. In tendon transfers, real-time movement aids precise tensioning [35,36]. Awake flap surgery is also feasible. Overall, WALANT spans nearly all hand surgeries, offering comparable or improved results with less anaesthetic risk.

#### 3.1.2. Ultrasound-Guided Nerve Block

Ultrasound-guided distal nerve blocks can offer a targeted, safe, and efficient anaesthetic option for elective and outpatient hand procedures. Blocking the median, ulnar, or radial nerves at the forearm provides reliable anaesthesia for surgeries like CTR, tendon repair, and Dupuytren’s surgery, without general anaesthesia or tourniquet use [37,38,39]. These blocks may outperform proximal approaches as they preserve proximal motor function, minimising systemic effects, and hastening postoperative recovery [38,39]. In contrast, proximal approaches can prolong postoperative recovery due to dense motor blockade of the entire upper limb, necessitating extended monitoring and limiting immediate functional use of the arm, factors that can delay discharge in ambulatory settings [39].

Moreover, in emergency settings, ultrasound-guided distal nerve blocks have been shown to offer rapid, opioid-sparing analgesia for acute injuries such as fractures and lacerations [40,41]. In children, blocks combined with light sedation allow for rapid and effective care while providing prolonged postoperative analgesia [41]. Furthermore, ultrasound-guided distal nerve blocks have consistently demonstrated greater efficacy and reliability than landmark-based techniques or simple local infiltration, including in some paediatric patients (where anatomic variability might be more pronounced). For example, a study by Liu et al. (2018) achieved a 98% success rate compared to 82% with landmark digital blocks in paediatric trigger-thumb procedures [42]. Furthermore, a meta-analysis by Guay et al. (2019) reported an 11% absolute reduction in peripheral nerve block failures in children when using ultrasound guidance compared to traditional blind techniques [43].

By enabling precise perineural injection of local anaesthetic in clinical or procedure-room settings, ultrasound-guided distal nerve blocks eliminate the necessity for formal operating theatre time and preoperative fasting associated with sedation-based techniques [44]. Since the technique is distal and precise, and as patients remain conscious, surgeons may be able to undertake interactive procedures, such as active tendon testing during repair, benefiting from real-time feedback, thereby enhancing accuracy and functional outcomes [45,46]. However, this is highly dependent on the location of the block, and in many cases, this is not feasible.

Furthermore, the targeted analgesia provided by ultrasound-guided distal nerve blocks has been extended to outpatient chronic pain management, including the alleviation of complex regional pain syndrome (CRPS), neuroma-related neuralgic pain, and other neuropathic conditions, wherein repeated clinic-based blocks can afford sustained symptom control with minimal systemic exposure [47,48]. This versatility underscores the role of ultrasound-guided distal nerve blocks in both acute procedural and long-term pain management, presenting a safe and cost-effective alternative to theatre-based or pharmacologically intensive methods.

The ultrasound-guided distal nerve blocks support five main clinical domains: elective surgery, emergency care, postoperative analgesia, outpatient procedures, and chronic pain management. Its safety, precision, and adaptability make it a valuable and expanding component of hand surgery anaesthesia.

#### 3.1.3. Emerging Adjuncts

##### Vapocoolant Spray

The most established use of vapocoolant sprays is in reducing pain during local anaesthetic injections, particularly digital nerve blocks. Applied 15–30 s before needle insertion, they significantly decrease discomfort from both puncture and infiltration. In a randomised trial, Selvi et al. (2021) reported 30–50% lower visual analogue scores in such cases [14]. They are also widely used for therapeutic injections in trigger finger, De Quervain’s tenosynovitis, and carpal tunnel release. While one trial reported no statistically significant pain reduction, a national survey found that 60% of hand surgeons use vapocoolants regularly for perceived patient comfort [49]. Their application has also expanded to ultrasound-guided injections, where patients often prefer spray over lidocaine wheals due to reduced skin distortion [50,51]. In paediatric and emergency settings, vapocoolants provide rapid, needle-free analgesia for procedures including nail repairs and fracture reductions, enhancing both comfort and procedural success [50].

Beyond injections, vapocoolants have been used in venous and arterial cannulation [50,51,52,53,54]. Zhu et al. (2018) demonstrated reduced intravenous insertion pain without affecting cannulation success [54], and Rüsch et al. (2017) found vapocoolants equivalent to lidocaine for radial artery access [55]. Vapocoolants are a simple, effective option for surface-level analgesia.

##### PCRA

PCRA is typically reserved for procedures associated with significant postoperative pain or for patients requiring prolonged analgesia [56,57]. Common indications include complex trauma, digital replantation, flap surgery, and fracture fixation, situations where opioids may provide insufficient post-operative pain relief [58,59]. The technique involves placing a perineural catheter adjacent to the target nerve (e.g., median, ulnar, or brachial plexus) and connecting it to a portable pump, allowing patient-controlled dosing. In a randomised trial, Gupta et al. (2011) demonstrated that PCRA with ropivacaine provided superior pain control and reduced opioid consumption following carpal tunnel release [60]. While outpatient use is increasing, the resource and expertise requirements currently restrict availability.

##### Cryoanalgesia

Cryoanalgesia is gaining traction for surgical and chronic pain relief [61,62,63,64,65]. It temporarily ablates sensory nerves via a cryoprobe, providing weeks of analgesia [62,64]. Intraoperative cryoneurolysis of the median, ulnar, or radial nerves has been used in contracture release and wrist surgery to prolong block duration [66]. It has also reduced phantom pain after brachial plexus cryoablation during amputation [66] and demonstrated durable results in digital neuromas and entrapments [67]. Additional uses include CRPS, post-traumatic neuralgia, and superficial tendinopathies like de Quervain’s or lateral epicondylitis [67,68]. Performed under ultrasound, it is safe, repeatable, and typically done in under 30 min.

##### Jet Injection

Jet injection offers a needle-free method for delivering local anaesthesia, using high-pressure bursts to transdermally administer lidocaine [69,70,71]. Commonly employed before corticosteroid injections for trigger finger, carpal tunnel release, or De Quervain’s tenosynovitis, it can improve patient comfort and reduce needle-related anxiety. In a prospective study, Patel et al. (2021) reported high patient satisfaction despite no statistically significant difference in pain scores compared with conventional injection [72]. Therefore, the observed benefit may relate to a placebo effect or reduced procedural anxiety, factors that can meaningfully influence patient experience, particularly in paediatric care.

### 3.2. Economic Implications: Cost-Effectiveness and Efficiency

#### 3.2.1. WALANT

WALANT offers significant cost advantages by eliminating the need for general anaesthesia, anaesthesia providers, and post-anaesthesia care. Rhee et al. (2017) reported nearly $4000 in savings per case within a U.S. military context, amounting to $393,100 across 100 procedures [4]. In Canada, Leblanc et al. (2007) found that carpal tunnel surgeries performed under WALANT in procedure rooms cost only one-third of those in main operating theatres using regional blocks [73]. Similarly, Maliha et al. (2019) in New York observed $3344 savings per trigger finger case using WALANT, with OR staffing alone costing $44 more per minute under conventional anaesthesia [74]. In Spain, Far-Riera et al. (2022) documented over $1000 saved per case for common procedures like carpal tunnel and trigger finger release [75].

Beyond per-case cost savings, WALANT may improve operational efficiency. By removing anaesthetic induction, airway management, and recovery phases, surgeons may be able to rotate seamlessly between procedure rooms [76]. The surgeon can administer the lidocaine-epinephrine at the start and wait 25–30 min for peak vasoconstriction and optimal haemostasis [77]. The surgeon can therefore potentially complete a WALANT case and immediately transition to the next room once the block takes full effect and top up if needed at that time [20]. This, in addition to minimal setup and turnover time, may provide a streamlined approach that facilitates genuine parallel or back-to-back scheduling across multiple rooms, thereby eliminating the usual anaesthesia-related delays and bottlenecks.

Finally, patients typically resume daily activities within 24 h, avoiding sedation-related recovery delays, reducing indirect patient costs from time off work. Though reimbursement structures may lag, the cumulative evidence across health systems supports WALANT as a high-value, cost-efficient model for hand surgery [74,75,76,77,78,79].

#### 3.2.2. Ultrasound-Guided Nerve Block

Ultrasound-guided distal nerve blocks are emerging as a cost-effective anaesthetic strategy in hand surgery. Compared to brachial plexus blocks or general anaesthesia, distal wrist and forearm blocks reduce drug use, setup time, and recovery demands [80]. Baradaran et al. (2023) reported a 46% reduction in anaesthesia-related costs when using local blocks ($236 vs. $435), driven by shorter anaesthesia times and reduced medication and monitoring needs [80]. Further, the time required to inject a distal block compared to a proximal block is usually shorter, with one study finding significant differences between the two, with 1.3 min for distal blocks versus 7.0 for plexus blocks, yielding notable OR time savings [80].

Workflow optimisation is another benefit. As distal blocks involve superficial nerves, they can be safely performed in procedure-room settings under minimal monitoring. Nijs et al. (2023) showed that using a block room for distal blocks reduced OR occupancy and anaesthesia-related delays [7]. Winter et al. (2020) found that procedures performed under ultrasound-guided blocks in minor rooms, rather than ORs, cut system costs and improved throughput [45]. In addition, distal blocks usually do not require post-anaesthesia care unit admission, and as described above, have no sedation-related side effects, hastening recovery.

While dedicated block rooms have proven effective for distal nerve blocks, their suitability for proximal techniques hinges on available resources and monitoring standards. Proximal approaches, such as interscalene or infraclavicular brachial plexus blocks, carry risks (e.g., phrenic nerve palsy, pneumothorax, systemic local-anaesthetic toxicity) that mandate continuous ASA-standard monitoring (including ECG, pulse oximetry, non-invasive blood pressure, and capnography when sedation is used) and immediate access to airway management and resuscitation equipment [81]. Unless a block room is staffed and equipped to these same specifications, including qualified anaesthesia personnel present throughout, proximal blocks are best performed in an operating theatre or a purpose-built block suite that meets full anaesthesia-care requirements [81].

Patients also benefit: regional blocks shorten hospital stays and enable a quicker return to normal activities, thereby lowering indirect costs such as lost work [82]. Although equipment and training carry upfront costs, the growing availability of affordable handheld ultrasound devices helps overcome these barriers [45].

Compared to WALANT, distal blocks may require more resources but offer better control for longer or more invasive procedures [83]. Ultrasound-guided distal nerve blocks provide a scalable, flexible, and economically viable anaesthetic option for modern hand surgery [82].

#### 3.2.3. Emerging Adjuncts

Emerging adjuncts such as vapocoolants, PCRA, cryoanalgesia, and jet injection technologies offer nuanced economic trade-offs in hand surgery.

##### Vapocoolant Spray

Vapocoolant sprays, such as ethyl chloride, are a low-cost, efficient intervention with wide applicability in hand surgery [54]. A single 103 mL bottle, costing only a few dollars, can last for weeks in a high-volume clinic. Sandrowski et al. (2021) reported an average usage of 1.9 mL/day, equivalent to just a few cents per patient [84]. Compared to options like EMLA cream or injectable lidocaine, vapocoolants offer immediate analgesia without the need for additional materials, preparation time, or staffing costs [54].

Clinically, they streamline workflow by eliminating lidocaine onset delays. Rüsch et al. (2017) demonstrated faster arterial cannulation with vapocoolant, while Page & Taylor (2010) noted staff preferred its simplicity over injectable setups [52,55]. Economically, the ability to reduce injection pain can obviate sedation in needle-phobic patients, avoiding OR-level resources for minor procedures. Though Franko and Stern (2017) found mixed results in certain applications, the spray’s low cost and high patient satisfaction support its ongoing use [49].

By improving comfort during anaesthesia, vapocoolants may increase acceptance of in-office procedures, complementing WALANT’s cost-saving model. Given their safety, speed, and positive patient experience, vapocoolants are a valuable tool in hand surgery preparation.

##### PCRA

PCRA can reduce hospitalisation costs by facilitating outpatient pain control. Yoo et al. (2025) in a review demonstrated that the use of peripheral nerve catheters resulted in a 2–3 day reduction in hospital stays, leading to significant savings in bed occupancy and nursing time [85]. However, PCRA carries upfront expenses, including catheter kits, pumps, staffing, and follow-up, which may not be cost-effective for minor cases. Selective use in complex procedures, where it replaces inpatient analgesia, offers the most favourable cost–benefit profile [86].

##### Cryoanalgesia

Cryoanalgesia also shows promise for reducing long-term costs by limiting opioid use and repeat interventions [87]. In CRPS or neuroma cases, a single session may supplant months of medication or serial nerve blocks [88]. While capital costs and variable reimbursement remain barriers, the potential for durable relief supports its targeted adoption.

##### Jet Injectors

Jet injectors, such as the J-Tip, add a modest per-use cost (~$30–$40) but may streamline care by avoiding sedation and improving patient cooperation, especially in pediatric or needle-phobic populations [19,89]. Though typically unreimbursed, they may indirectly reduce ancillary costs and support adherence to nonoperative interventions. Across these modalities, careful patient selection remains central to ensuring economic viability.

### 3.3. Limitations and Contraindications

#### 3.3.1. WALANT

While WALANT has revolutionised hand surgery, it is not universally applicable. The only absolute contraindication is allergy to local anaesthetics, such as lidocaine, a rare but definitive exclusion [20]. Patient refusal is another absolute barrier, as cooperation is essential during awake procedures [90,91].

Several relative contraindications merit caution. Patients with severe anxiety, psychiatric illness, or needle phobia may not tolerate the experience, even when pain is minimal [81]. While mild anxiety is often manageable, more severe cases may benefit from sedation or general anaesthesia. In paediatric cases, especially with younger children, the need for sedation limits WALANT’s role.

Vascular insufficiency is a key concern. Conditions like Raynaud’s disease, Buerger’s disease, or recent digital replantation may be worsened by epinephrine-induced vasoconstriction [21,91]. Patients with sickle cell disease also face the risk of vasoocclusive crises under reduced perfusion [91]. Coagulopathy and anticoagulant therapy pose challenges: without a tourniquet, bleeding control is more difficult, and uncorrected clotting disorders increase haematoma risk despite vasoconstriction [91].

Procedural factors also influence suitability. Surgeries lasting beyond 2–3 h, or involving proximal limb regions, may exceed anaesthetic coverage or involve referred pain beyond the infiltrated zone [92]. Movement disorders, cognitive impairment, or poor cooperation may destabilise patient positioning during critical steps. Finally, operator inexperience or inadequate guidance may lead to block failure, potentially requiring conversion to general anaesthesia [92,93]. In summary, WALANT is safe and effective when applied judiciously, with appropriate patient selection, procedural matching, and operator proficiency.

#### 3.3.2. Ultrasound-Guided Nerve Block

Despite their growing utility, ultrasound-guided distal nerve blocks have inherent limitations. Most notably, their anaesthetic range is restricted to the distal forearm and hand, making them unsuitable for proximal procedures. Additionally, standard upper-arm tourniquets remain painful due to unblocked intercostobrachial innervation, limiting their use without supplemental anaesthesia [38]. Forearm tourniquets can alleviate this but are constrained in both duration and tolerance [94].

Incomplete anaesthesia is a recognised issue. Anatomical variations, such as high division of the median nerve or missed branches like the anterior interosseous, can result in patchy blocks. One study found that 15% of patients required additional infiltration despite forearm blocks [38]. Visualising smaller nerves (e.g., the radial nerve) can be technically challenging for novices [95], and institutions transitioning from proximal blocks must account for this learning curve and outcome variability.

General contraindications include local infection, coagulopathy, and pre-existing neuropathy. Though distal blocks may be safer in anticoagulated patients due to the compressibility of the forearm [83], vigilance remains necessary. Patient-related factors, high anxiety, young age, or cognitive impairment may necessitate sedation. Repeated injections increase the risk of haematoma or local anaesthetic systemic toxicity (LAST), particularly if aspiration is not performed between doses [45].

In summary, while offering clear advantages over proximal techniques, ultrasound-guided distal nerve blocks must be applied thoughtfully, with close attention to anatomy, technical execution, and patient selection.

#### 3.3.3. Emerging Adjuncts

Adjuncts such as vapocoolant spray, PCRA, cryoanalgesia, and jet injectors provide expanded options but come with specific limitations.

##### Vapocoolant Spray

Vapocoolant sprays provide brief, superficial analgesia, limiting their role as a primary anaesthetic. Their effect lasts only seconds and reaches only the epidermis and superficial dermis, rendering them unsuitable for deeper procedures involving tendon or bone [96,97]. Their ideal use remains as adjuncts during local anaesthetic infiltration or brief superficial procedures.

Cooling-induced vasoconstriction raises risks in patients with compromised circulation. In those with Raynaud’s phenomenon, peripheral arterial disease, or recent revascularisation, the transient reduction in blood flow could provoke ischaemia [96,97]. Frostbite or skin injury can occur if the spray is applied too closely, for too long, or over compromised tissue. Application should be limited to intact skin, with use over wounds or mucosal surfaces strictly avoided. Ethyl chloride’s flammability poses a risk near ignition sources or electrocautery [98,99], while inhalation exposure, though rare, may be problematic in patients with arrhythmias or cardiopulmonary conditions [96,97,98]. Dermatologic reactions such as cold urticaria or contact dermatitis are absolute contraindications [97].

Some patients may dislike the cold sensation or aerosol noise, particularly children or those with anxiety. However, correct technique, brief spraying from 12–18 cm onto dry skin, can optimise comfort and safety [98]. In summary, vapocoolants are low-risk and effective when used appropriately; however, their narrow analgesic window and vasoconstrictive effects necessitate careful patient and procedural selection.

##### PCRA

PCRA requires catheter placement, an infusion pump, and active patient cooperation. It is contraindicated in individuals with cognitive impairment, non-compliance, bleeding disorders, or local infection at the insertion site [99,100]. Though rare, PCRA remains a concern if dosing exceeds recommended limits [99]. PCRA is not a substitute for intraoperative anaesthesia and may mask compartment syndrome, particularly in trauma patients, making this a key contraindication.

##### Cryoanalgesia

Cryoanalgesia, while useful for extended pain relief, should be avoided in patients with cold sensitivity (e.g., Raynaud’s syndrome, cryoglobulinemia), open wounds, or active infections [101]. It is also contraindicated for nerves with essential motor function due to the risk of transient paresis. For example, cryoneurolysis targeting the musculocutaneous nerve branch to the biceps or brachialis may impair elbow flexion during the regenerative phase [102]. Additional limitations include the requirement for specialised equipment and precise nerve access techniques. Patients should be counselled about the possibility of neuropathic symptoms during nerve regeneration, including sensory dysesthesia or paraesthesia.

##### Jet Injectors

Jet injectors, though needle-free and appealing for superficial anaesthesia, are limited by depth and volume. They are ineffective for more profound or extensive anaesthesia and require proper technique for safe use [103,104]. Relative contraindications include inflamed skin, lidocaine allergy, or extreme needle/noise aversion [105]. Misuse can lead to incomplete anaesthesia or bruising, especially in anticoagulated patients. Additionally, jet injectors have a short duration of effect and are not suitable for lengthy procedures.

In summary, while these adjuncts enhance the anaesthetic toolbox in hand surgery, careful patient selection and procedural judgment are essential to avoid risks and optimise outcomes.

To better summarise the utility of the discussed anaesthesia techniques, Table 1 provides a concise comparison of each anaesthetic modality’s analgesic and motor effects, operative field characteristics, intraoperative testing capabilities, and key workflow implications to guide technique selection.

The optimal anaesthetic technique depends on surgical goals. If intraoperative testing is needed, WALANT is ideal. If a bloodless field is the priority and no testing is required, a regional block with a tourniquet may be the preferred approach. Many procedures lie between these extremes, requiring the surgeon to weigh functional needs, patient factors, and resource availability.

Rigid adherence to one method can be counterproductive. For example, insisting on WALANT in highly anxious or needle-phobic patients may cause undue distress, while defaulting to sedation in cooperative patients can undermine WALANT’s efficiency and cost benefits. Tailoring the technique to each patient optimises outcomes and minimises risk.

Decision-making must also include contingency planning. If WALANT fails due to unexpected pain or anxiety, sedation or conversion to a block, or even general anaesthesia, should be readily available. Conversely, if a regional block proves incomplete (with failure rates reported at 5–10%), supplementary local anaesthetic can convert the case to a WALANT-like approach or facilitate swift escalation to GA. Flexibility is essential for safe and responsive intraoperative care.

The matrix (Table 2), though simplified, demonstrates that no single technique is suitable for all scenarios. Instead, decisions should be individualised, often combining modalities for the best effect. For example, a jet injector can make a WALANT injection more tolerable, or a regional block may be paired with patient-controlled sedation to manage both pain and anxiety.

This matrix (Table 2), though simplified, demonstrates that no single technique is suitable for all scenarios. Instead, decisions should be individualised, often combining modalities for the best effect. For example, a jet injector can make a WALANT injection more tolerable, or a regional block may be paired with patient-controlled sedation to manage both pain and anxiety.

## 4. Conclusions

The evidence presented in this review indicates that WALANT and ultrasound-guided distal nerve blocks each offer tangible benefits in ambulatory hand surgery, namely, reduced per-case costs, shorter turnaround times, and preservation of functional testing, when compared with traditional general anaesthesia or proximal regional techniques. Adjunctive strategies, such as vapocoolant sprays and jet injectors, can improve patient comfort during injections. In more complex cases, cryoanalgesia and patient-controlled regional analgesia offer targeted postoperative pain relief. In practical terms, incorporating WALANT or distal blocks as first-line options can streamline workflow and improve patient experience in office-based or minor procedures. Adjuncts should be selected selectively to address specific procedural or patient-centred needs (e.g., needle phobia or prolonged analgesia) rather than being used universally. However, clear guidance on the best technique combinations is limited by a lack of head-to-head economic comparisons, long-term functional outcome data, and standardised adjunctive protocols. Future prospective trials comparing these methods directly, and assessing their impact on recovery, functional outcomes, and cost-effectiveness across different healthcare settings, are necessary to develop evidence-based anaesthesia algorithms in hand surgery.

## Figures and Tables

**Table 1:** Utility of hand surgery anaesthesia techniques.

Technique	Analgesia & Motor Block	Field Conditions	Intraoperative Testing	Time Constraints & Workflow
**WALANT**	Complete sensory block No motor block once the local wears off	Mild bleeding (epi-assisted) No tourniquet required	Active motion testing (tendon/glide)	No time limit Quick prep (5–10 min)
**Ultrasound-Guided Block**	Complete sensory & motor block	Bloodless with a tourniquet	Cannot test active movement	Prep ∼5–15 min No intraoperative interruptions
**Vapocoolant/Jet Injector**	Brief, superficial analgesia for needle pain only	N/A (adjunctive)	N/A	Instant onset Minimal time added
**Patient-Controlled Sedation**	Supplement to local/regional block	As per the underlying block/LA	As per the underlying block/LA	Allows self-titration for procedure spikes
**Cryoneurolysis**	Prolonged sensory blockade, no motor blockade	N/A (adjunctive)	N/A	Setup ~10 min; outpatient sessions; durable relief

Abbreviations: LA = local anaesthetic, N/A = not available, WALANT = wide-awake local anaesthetic no tourniquet.

**Table 2:** Decision matrix for anaesthesia in hand surgery.

Scenario/Factor	WALANT	Ultrasound Guided Nerve Block	Supplemental Emerging Adjuncts
**High patient anxiety or fear**	Not ideal without additional support	Suitable with light sedation	PCRA: suitable if patient understands it
**Need for active movement testing**	Ideal	Not suitable (motor block)	No direct effect
**Requirement for tourniquet use**	Less Ideal	Ideal (blocks cover tourniquet pain)	No direct effect
**Short operation (<30 min)**	Ideal	Less efficient (block setup time)	Jet/vapocoolant: time-saving adjunct
**Long operation (>2 h)**	Possible with reinjection risk	Ideal (long-acting agents)	Cryoneurolysis: adjunct for prolonged analgesia
**Extensive surgical field**	Less ideal (large volume needed)	Ideal (plexus coverage)	No primary effect
**Avoidance of general anaesthesia**	Ideal	Ideal	Adjuncts enhance other techniques
**Anticoagulation present**	Suitable	Depends on the block level	Jet/vapocoolant: suitable; cryoneurolysis cautious
**Uncooperative**	Not suitable	Not suitable	No substitute for GA in children
**No anaesthetist available**	Ideal	Ideal	Jet/vapocoolant: suitable
**Office/clinic setting**	Ideal	Rarely used	Jet/vapocoolant: ideal; PCRA not without monitoring

Abbreviations: GA = general anaesthesia, PCRA = patient-controlled regional anaesthesia, WALANT = wide-awake local anaesthetic no tourniquet.

## Abbreviations

The following abbreviations are used in this manuscript:

GA	General Anaesthetic
IVRA	Intravenous Regional Anaesthesia
LA	Local Anaesthetic
PCRA	Patient Controlled Regional Anaesthesia
WALANT	Wide Awake Local Anaesthesia No Tourniquet
CRPS	Complex Regional Pain Syndrome
